# Genes Involved in Lipid, Carbohydrate, and Protein Metabolism as Candidates Affecting Beef Flavor

**DOI:** 10.3390/ani16071003

**Published:** 2026-03-25

**Authors:** Andrea Rando, Giulia Grassi, Anna Maria Perna, Paola Di Gregorio

**Affiliations:** Dipartimento di Scienze Agrarie, Forestali, Alimentari ed Ambientali, University of Basilicata, Via dell’Ateneo Lucano 10, 85100 Potenza, Italy; andrea.rando@unibas.it (A.R.); giulia.grassi92@yahoo.it (G.G.); anna.perna@unibas.it (A.M.P.)

**Keywords:** cattle, beef flavor, Quantitative Trait Loci, candidate genes

## Abstract

Beef flavor is a trait difficult to evaluate since different senses (taste, touch, and smell) are involved in its perception. In the last 20 years, 102 Quantitative Trait Loci (QTLs), associated with the variability of different beef flavor notes, have been reported. These QTLs are spread on all chromosomes, including BTA X. In these QTL regions, 2509 genes are located and, among them, 594 are involved in the metabolic processes of lipids, proteins, and carbohydrates, the main meat components for the production of volatile substances responsible for flavor. Only 19 of these genes (*ACSM2B*, *ACSM3*, *ACSM4*, *ACSM5*, *CHID1*, *DHCR7*, *EDEM3*, *GDE1*, *HEXB*, *IGF2*, *INS*, *NDUFAB1*, *PIGC*, *PNPLA2*, *PRDX6*, *SCNN1B*, *SIAE*, *SMG1*, and *UMOD*) are also present in the QTL regions affecting pork flavor. The applied approach allowed us to strongly restrict the number of candidate genes to affect the variability of both beef and pork flavor.

## Simple Summary

Meat flavor, perceived through different senses such as taste, touch, and smell, is a key factor in consumer choices. It is often considered as an indicator of meat quality, since the volatile compounds that develop during cooking are the results of chemical reactions involving meat components (lipids, proteins, and carbohydrates). Meat flavor is affected by several environmental and genetic factors with the consequence that its definition is rather complex. In this paper, we identified 19 candidate genes located in both cattle and pig flavor Quantitative Trait Loci (QTL) regions and involved in the metabolic processes of lipids, proteins, and carbohydrates. The analysis of these genes could shed light on meat flavor variability.

## 1. Introduction

According to the International Standardization Organization [1], flavor can be described as a “complex combination of the olfactory, gustatory, and trigeminal sensations perceived during tasting”. Flavor, together with tenderness and juiciness, is one of the attributes affecting beef consumer eating satisfaction and is usually used to assess meat quality. In recent years, flavor has assumed a greater weight in the meat acceptability rate since selection activity has allowed us to obtain good values for beef tenderness [2,3,4,5].

Several environmental factors, including feeding, slaughter age, pre-slaughter and postmortem factors, aging, marination, and cooking conditions, are able to influence beef flavor [6,7,8,9]. In particular, cooking is the step responsible for the development of volatile compounds which arise from heat-triggered reactions: lipids undergo oxidation with the production of aldehydes, alcohols, and ketones; proteins are reduced to peptides and amino acids which, in the presence of reducing sugars, give intermediate compounds such as furanoids, pyrroles, pyridines, and pyrazines (Maillard reaction); amino acids undergo Stecker degradation, contributing to the formation of pyrazines; thiamine (vitamin B1) degradation produces a series of sulfur-containing compounds. Furthermore, the products obtained from the individual reactions can interfere with each other by blocking the production of some compounds or interact to form new ones [3,10]. It follows that flavor is strongly dependent on the quantity and quality of carbohydrates, proteins, and lipids characterizing the variability of meat quality.

Studies on the genetic variability of beef flavor notes refer only to QTLs identified in different cattle breeds by using mainly panel tests. As far as we are concerned, no causative mutation responsible for flavor variability has been reported. In pigs, the analysis of genes present in the 99 QTL regions associated with the variability of pork flavor allowed us to identify 107 genes, out of about 3000, that are involved in lipid, carbohydrate, lipoprotein, and glycoprotein metabolic processes [11].

The aim of this work was to identify candidate genes for beef flavor by means of: (a) listing all the genes present in the beef flavor QTL regions; (b) restricting this number to genes that are involved in lipid, protein, and carbohydrate metabolic processes; and (c) making a comparison between these genes and the ones obtained in the same way in pigs.

## 2. Materials and Methods

The Cattle QTL Database (release 57) (https://www.animalgenome.org/cgi-bin/QTLdb/BT/index, accessed on 23 October 2025) was analyzed to identify bovine QTLs affecting beef flavor traits (overall impression, meat flavor score, juiciness, beef flavor intensity, abnormal flavor intensity, beef odor intensity, and abnormal odor intensity) belonging to Sensory Characteristics of Meat and Carcass Traits [12]. The position of the markers associated with each QTL was checked by referring to the Ensembl genome browser (release 115) (https://www.ensembl.org/index.html, accessed on 10 November 2025) for Single Nucleotide Polymorphism (SNP)-like markers [13]. In addition, the positions of primers used to analyze microsatellite regions and of markers within gene sequences were verified by referring to the *Bos taurus* assembly ARS-UCD2.0 (GCF_002263795.3) (https://www.ncbi.nlm.nih.gov/gdv/browser/genome/?id=GCF_002263795.3, accessed on 10 November 2025).

The same assembly was used to identify genes located within the flavor QTLs after defining the search ranges from the upper to the lower marker for each QTL or from 1 Million base pair (Mbp) upstream to 1 Mbp downstream for QTLs with a known, well defined peak marker.

Gene Ontology (GO) analysis of the genes located within the flavor QTL regions was performed by using the DAVID Knowledgebase v2023q4 (https://davidbioinformatics.nih.gov/, accessed on 7 January 2026) [14,15]. Significance thresholds for GO analysis were as follows: a maximum probability *p*-value ≤ 0.05 and a minimum gene count for an annotation term ≥2.

## 3. Results and Discussion

At present, 102 QTLs affecting beef flavor are reported in the cattle QTL database. Only two of these QTLs are located on BTA X; the others are distributed on all autosomal chromosomes with a maximum of 10 on BTA 7 and a minimum of 1 on BTAs 11, 24, and 26. Beef flavor QTLs are classified into seven notes/traits: overall impression (4 QTLs), meat flavor score (13 QTLs), juiciness (65 QTLs), beef flavor intensity (8 QTLs), abnormal flavor intensity (4 QTLs), beef odor intensity (3 QTLs), and abnormal odor intensity (5 QTLs) (Table S1) [16,17,18,19,20,21,22,23,24,25,26,27,28,29] and have been identified in different breeds or crosses (Table S2) [16,17,18,19,20,21,22,23,24,25,26,27,28,29].

By means of the analysis of *Bos taurus* assembly ARS-UCD2.0 (GCF_002263795.3), 2509 genes in the autosomal QTL regions were identified (Table S3). Gene Ontology (GO) analysis, performed by using the DAVID Knowledgebase v2023q4, allowed us to restrict this number to 594 genes significantly involved in processes concerning the metabolism of proteins, lipids, and carbohydrates—that is, the main components of the chemical reactions (Maillard reaction, Stecker degradation, and lipid oxidation) affecting meat flavor (Table 1).

In order to further restrict the number of candidate genes affecting flavor, we compared genes shown in Table 1 with those reported for pork QTL flavor by Di Gregorio et al. [11] highlighting only 19 genes in common. Since these 19 genes are both located in QTL regions affecting beef and pork flavor and involved in protein, lipid and carbohydrate metabolic processes (see Table 1 and Table 2) it can be consequently hypothesized that they are strong candidates to affect the variability of meat flavor. This consideration is further supported by the fact that, as shown in Table 2, these genes are located in QTL regions affecting similar or identical flavor notes in two species belonging to distinct Artiodactyla suborders (Ruminantia vs. Suiformes) whose meats are characterized by clear-cut differences (for example: protein, fat, vitamin and minerals content; fat composition) [30,31,32].

Proteins produced by *ACSM2B*, *ACSM3*, *ACSM4,* and *ACSM5* genes are involved in acyl-CoA metabolic and fatty acid (FA) biosynthetic processes. The first step in the synthesis of all lipids, both structural ones, such as phospholipids or sphingolipids, and storage ones, such as triacylglycerol and cholesteryl esters, is the activation of FAs with coenzyme A (CoA) catalyzed by an acyl-coenzyme A synthetase (ACS). In higher organisms, there are several enzymes with this activity, which are classified according to the length and saturation level of the FA chain on which they mainly act: short-chain (ACSS), medium-chain (ACSM), long-chain (ACSL), and very long-chain (ACSVL) [33,34]. The four abovementioned synthetases typically act on medium-chain (in general, C6-C10) FA with a preference for isobutyrate (ACSM3), C6-12 FA (ACSM4), and C4-C10 FA (ACSM2B). In cattle, variations in acyl-coenzyme A synthetases acting on long-chain fatty acids have been associated with the FA composition of skeletal muscle (ACSL1) [35] and triglyceride metabolism (ACSL5) [36].

PIGC encodes one of the proteins of the glycosylphosphatidylinositol-N-acetylglucosaminyltransferase (GPI-GnT) complex involved in the first step of GPI lipid anchor biosynthesis [37]. The FA content of the anchor contributes to the lipid composition of the membrane and can determine the membrane-packing characteristics of the protein. In humans, different levels of expression of this gene are associated with the regulation of body fat distribution [38].

The *EDEM3* gene codes for a protein involved in endoplasmic reticulum-associated degradation (ERAD) ensuring that only properly folded proteins are retained in the cell. It may also participate in mannose trimming from all glycoproteins. In humans, loss of EDEM3 enzymatic activity determines a congenital disorder of protein glycosylation [39]. In cattle, variability in this gene has been associated with rib eye area [40].

The *PRDX6* gene encodes a bifunctional enzyme, a member of the thiol-specific antioxidant protein family, with peroxidase and phospholipase activity. As a consequence, this protein is involved both in cell protection against oxidative stress and phospholipid turnover [41,42]. In pigs, two polymorphisms in the coding region of this gene were associated with intramuscular fat variation [43].

The *HEXB* gene codes for the beta subunit of the lysosomal beta-hexosaminidase enzymes: Hex B (composed of two beta subunits), Hex A (composed of one alpha and one beta subunit), and Hex S (composed of two alpha subunits). The two subunits, encoded by separate genes, are members of family 20 of glycosyl hydrolases [44]. Both Hex A and B enzymes are involved in the catabolism of glycoproteins, glycosaminoglycans, and glycolipids [45]. In humans, mutations in the *HEXB* gene cause the onset of Sandhoff disease due to the reduced activity of both the Hex A and Hex B enzymes [46]. In the cattle HEXB locus, an EcoRI restriction fragment length polymorphism was reported in Brown Swiss and Simmental breeds [47].

NDUFAB1 encodes one of the 45 subunits that compose Complex I (NADH:ubiquinone oxidoreductase), the first enzyme of the mitochondrial respiratory chain, and it is the subunit essential for cell viability [48]. It is involved in FA biosynthesis. Overexpression of this gene protects mice against obesity and insulin resistance by promoting the oxidation of FA and, therefore, preventing their storage in adipocytes [49]. In cattle, the FA oxidation due to the activation of NDUFAB1 reduces the cytotoxic effects of high non-esterified fatty acid (NEFA) concentrations in adipocytes [50]. In chickens, NDUFAB1 has been identified as a possible candidate gene for intramuscular fat (IMF) deposition [51].

UMOD encodes the most abundant protein in mammalian urine under physiological conditions. Its excretion is possibly associated with the defense against urinary tract infections and kidney stone formation [52]. In humans, mutations of the UMOD promoter are associated with a reduced level of protein and an increased risk of chronic kidney disease (CKD) [53]. The onset of such diseases is associated with a strong alteration of lipid and lipoprotein metabolism [54,55].

DHCR7 encodes an enzyme involved in one of the final steps of cholesterol synthesis. In the Kandutsch–Russell pathway, it acts as a switch between cholesterol and vitamin D synthesis [56]. In goats, the *DHCR7* gene is involved in fat deposition, showing, on one hand, a negative effect on subcutaneous adipogenesis and, on the other hand, a positive effect on intramuscular adipogenesis [57].

IGF2 encodes a polypeptide growth factor of the insulin family that is involved in development and growth. It is engaged in the regulation of lipid metabolism [58], and its variability is associated with effects on several traits such as body mass index (BMI) in humans [59], FA composition in pigs [60], milk fat and protein content [61], and body weight in cattle [62].

The *INS* gene codes for the protein considered as the main regulator of carbohydrate, protein, and lipid metabolism due to its control activity on the normal plasma glucose homeostasis [63].

The *PNPLA2* gene produces a lipase that, through triglyceride hydrolysis, is involved in fat mobilization and lipid storage [64]. In cattle, two missense mutations identified in this gene were significantly associated with backfat (BF) thickness, dressing percentage, and marbling score [65]. In pigs, PNPLA2 polymorphisms were associated with BF thickness and FA composition [66].

It can be seen that many of the genes shown in Table 2 are associated with differences in the quantity and/or quality of meat fat. This result is in agreement with well-known data showing that, for example, the percentage of IMF is associated with the intensity of flavor and that a high level of PUFAs can negatively affect flavor [67,68]. Although fat is an essential solvent and precursor of volatile compounds [69], it must be kept in mind that flavor is a complex trait, the result of several factors that influence the development of volatile compounds from the muscle components (total fat, proteins, carbohydrates, vitamins, minerals, etc.) after cooking. Therefore, the analysis of the genes reported in this paper can only be the first step, to be followed by a multi-omics approach, to identify the mechanisms underlying flavor variability.

## 4. Conclusions

In this study, we identified 2509 genes located in the 102 QTL regions associated with beef flavor and, by means of Gene Ontology (GO) analysis, 594 genes involved in the metabolic processes of lipids, proteins, and carbohydrates, the main components from which volatile compounds responsible for flavor are developed. The comparison between the flavor QTL genomic regions of both cattle and pigs allowed us to restrict the number of genes strongly candidate to affect the variability of meat flavor to 19. Most of these genes are engaged in the metabolism of lipids, which play a crucial role in the formation of volatile compounds. The evaluation of the phenotypic effects of the variability of these 19 genes should be the first step to shed light on the genetic basis of meat flavor.

As a final consideration, the identification of shared genes in QTL regions for the same trait in two or more species can be considered an effective approach to restrict the number of candidate genes affecting the variability of any trait.

## Figures and Tables

**Table 1:** Genes located within beef flavor QTL regions significantly ascribed to lipid, carbohydrate, and protein metabolic processes. Genes also present inside pork flavor QTL regions are in bold and highlighted in red.

GO-Terms	Genes	*p*-Value
GO:0019538 Protein metabolic process (N. 437)	*AARS2*, *ABCB11*, *ABL2*, *ACE*, *ACE3*, *ACTMAP*, *ADAM11*, *ADAM17*, *ADAMTS14*, *ADAMTS15*, *ADAMTS18*, *ADAMTS8*, *ADAMTS9*, *ADCK1*, *AHSA1*, *AIMP2*, *AKT2*, *AKT3*, *ALG11*, *ALG5*, *ALKBH4*, *ALPK1*, *ANAPC15*, *ANAPC16*, *AP5Z1*, *ARL2*, *ARRDC4*, *ASPH*, *ASRGL1*, *ATG10*, *B3GALT6*, *B3GAT3*, *B3GLCT*, *B4GAT1*, *BAZ1B*, *BBS5*, *BFAR*, *BMPR1A*, *BPNT2*, *BRSK1*, *BRSK2*, *CAMK2D*, *CAPN1*, *CAPN11*, *CAPN12*, *CAPN2*, *CAPN8*, *CARS1*, *CASP9*, *CCDC47*, *CD6*, *CDC27*, *CDC42BPA*, *CDC42BPG*, *CDK11B*, *CDK5*, *CDK5RAP3*, *CELA2A*, *CHML*, *CHST3*, *CHST6*, *CLCA1*, *CLCA2*, *CLCA3*, *CLCA4*, *COP1*, *COPS5*, *CPA6*, *CPB2*, *CPE*, *CRYAA*, *CSTL1*, *CTRB2*, *CTRC*, *CTSD*, *CTSF*, *CTSW*, *CTTNBP2NL*, *CUL1*, *CUL7*, *CUL9*, *DAB2IP*, *DARS2*, *DCLK1*, *DCUN1D3*, *DDB1*, *DDI2*, *DERL1*, *DESI2*, *DLEU7*, *DNAJC10*, *DPP3*, *DTX2*, *DTX3L*, *DUOXA2*, *DUSP10*, *DUSP8*, *DYRK1B*, *EARS2*, ***EDEM3***, *EEF1G*, *EEF2K*, *EEFSEC*, *EGLN2*, *EIF1AD*, *EIF2AK1*, *EIF4H*, *ENC1*, *EOGT*, *EPHA8*, *EPHB2*, *ERAP1*, *ERAP2*, *ERN2*, *F13A1*, *FAM20B*, *FARS2*, *FAU*, *FBLN1*, *FBXL18*, *FBXO17*, *FBXO2*, *FBXO28*, *FBXO32*, *FBXO4*, *FBXO44*, *FBXO6*, *FBXW7*, *FKBP2*, *FKBP6*, *FLT1*, *FLT3*, *FNTB*, *GALNTL6*, *GAN*, *GANAB*, *GATB*, *GCSH*, *GFM2*, *GLUL*, *GOLGA7*, *GP5*, *GP9*, *GRK2*, *GTPBP2*, *HARS1*, *HARS2*, ***HEXB***, *HIPK1*, *HIPK4*, *HMCES*, *HS2ST1*, *HS3ST2*, *HSP90AB1*, *HSPA2*, *HSPB1*, *HSPH1*, *ICMT*, *IKBKB*, *ISG15*, *ITGB3*, *IVNS1ABP*, *KARS1*, *KAT5*, *KBTBD12*, *KBTBD6*, *KBTBD7*, *KBTBD8*, *KDM2A*, *KLHDC3*, *KLHL17*, *KLHL2*, *KLHL20*, *KLHL21*, *KLHL23*, *KLHL38*, *KLHL41*, *LEP*, *LIMK1*, *LMTK2*, *LMTK3*, *LNPEP*, *LRIG2*, *LRRC47*, *LYN*, *LYPLA1*, *LYPLA2*, *MACROD1*, *MAP3K10*, *MAP3K11*, *MAP3K14*, *MAP3K2*, *MAP3K20*, *MAP3K3*, *MAP4K2*, *MARK2*, *MASP2*, *MBTPS1*, *METAP1D*, *METTL18*, *MGC157405*, *MGC157408*, *MIB2*, *MMEL1*, *MMP23*, *MOS*, *MRPL10*, *MRPL11*, *MRPL14*, *MRPL15*, *MRPL2*, *MRPL20*, *MRPL21*, *MRPL23*, *MRPL45*, *MRPL49*, *MRPS12*, *MRPS14*, *MRPS18A*, *MRPS28*, *MTIF3*, *MTOR*, *MTRF1*, *MYO3B*, *MYRF*, *NAA16*, *NAA20*, *NAALADL1*, *NCCRP1*, *NEK3*, *NEK5*, *NFE2L1*, *NHLRC3*, *NIM1K*, *NMT1*, *NPEPPS*, *NPPA*, *NPPB*, *NR1D1*, *NSMCE2*, *NTAN1*, *NTAQ1*, *NTMT2*, *NUDCD2*, *NUP98*, *OTUB1*, *PAG10*, *PAG11*, *PAG12*, *PAG14*, *PAG15*, *PAG16*, *PAG17*, *PAG18*, *PAG19*, *PAG2*, *PAG20*, *PAG21*, *PAG3*, *PAG4*, *PAG5*, *PAG6*, *PAG7*, *PAG8*, *PAG9*, *PAK4*, *PAN3*, *PAPPA2*, *PARP1*, *PARP14*, *PARP9*, *PCMTD1*, *PCSK1*, *PDIA4*, *PDILT*, *PDK1*, *PELI3*, *PGA5*, *PGAP2*, *PIDD1*, *PIGC*, *PLAT*, *PLEKHN1*, *PLK1*, *PLOD1*, *POMT2*, *PPIG*, *PPM1J*, *PPP1CA*, *PPP2R5B*, *PPP2R5D*, *PRKCG*, *PRKCZ*, *PRKDC*, *PRMT3*, *PROC*, *PRPF19*, *PRPF4B*, *PRSS35*, *PSEN2*, *PSMA6*, *PSMC4*, *PSMC5*, *PSMD6*, *PTGES3*, *PTK7*, *PTPN14*, *PTPN20*, *PTPN21*, *PTPN22*, *PTPN5*, *PTPRH*, *QSOX1*, *RARS1*, *RBM4*, *RBP3*, *RC3H1*, *RCE1*, *RELA*, *RHOBTB3*, *RIOK2*, *RNASEL*, *RNF121*, *RNF139*, *RNF144A*, *RNF146*, *RNF170*, *RNF2*, *RNF216*, *RNF223*, *RNF41*, *ROCK2*, *RPL11*, *RPL18*, *RPL21*, *RPL22*, *RPL28*, *RPLP2*, *RPN1*, *RPS15A*, *RPS16*, *RPS20*, *RPS23*, *RPS6KA4*, *RPS6KB2*, *SAE1*, *SARS2*, *SBK2*, *SBK3*, *SCRN2*, *SCYL1*, *SCYL3*, *SDE2*, *SDHAF2*, *SGK3*, *SH3GLB1*, *SHMT2*, *SIAH3*, *SIK1*, *SIRT2*, *SLC35B2*, *SLC35C2*, ***SMG1***, *SMYD2*, *SOCS6*, *SOCS7*, *SPATA5L1*, *SPPL2C*, *SSC5D*, *SSH3*, *ST14*, *ST8SIA4*, *STK39*, *STRADA*, *STX1A*, *STYXL1*, *SUCO*, *SULF2*, *SYVN1*, *TARDBP*, *TBX21*, *TCIRG1*, *TLK2*, *TLL1*, *TMEM258*, *TMUB1*, *TOLLIP*, *TP53RK*, *TRIB1*, *TRIB2*, *TRIM13*, *TRIM2*, *TRIM50*, *TRIM55*, *TRIM58*, *TRMT112*, *TRPM4*, *TSG101*, *TSPAN33*, *TTBK1*, *TTLL10*, *TTLL11*, *TYSND1*, *UBA3*, *UBE2E3*, *UBE2J2*, *UBE2S*, *UBE2V2*, *UBE3D*, *UBR3*, *UBXN2B*, *UEVLD*, *UFM1*, ***UMOD***, *UQCRC2*, *USP12*, *USP31*, *USP42*, *USPL1*, *VASH1*, *VCPIP1*, *VIPAS39*, *VPS36*, *VPS37C*, *VPS37D*, *VSIR*, *VWA1*, *WDR26*, *WDR77*, *WIPI2*, *XYLT1*, *ZAR1L*, *ZDHHC13*, *ZDHHC22*, *ZDHHC4*, *ZDHHC5*, *ZNRF1*	1.65 × 10^−7^
GO:0006629 Lipid metabolic process (N. 145)	*ABCB11*, *ABHD11*, *ACAD9*, *ACBD3*, *ACBD4*, *ACOT12*, *ACOT7*, *ACSM1*, ***ACSM2B***, ***ACSM3***, ***ACSM4***, ***ACSM5***, *ADHFE1*, *ALDH3B1*, *APOF*, *APON*, *ATP5F1B*, *BAX*, *BBS1*, *BCAT2*, *BCO1*, *BLOC1S6*, *BPNT2*, *BSCL2*, *CERK*, *CERKL*, *CERS6*, *CHKA*, *CPT1A*, *CYP27C1*, *CYP2A13*, *CYP2B39*, *CYP2B6*, *CYP2F1*, *CYP2S1*, *CYP7A1*, *DAGLA*, *DAGLB*, *DBI*, *DEGS1*, *DGKH*, ***DHCR7***, *DHRS3*, *DHRS9*, *DISP3*, *EBPL*, *ECH1*, *ECHDC1*, *EPHX1*, *FADS1*, *FADS2*, *FADS3*, *FNTB*, *FUCA1*, *FUT1*, *FUT2*, *GAL3ST3*, *GALC*, ***GDE1***, *GPAT4*, *GSTP1*, ***HEXB***, *HMGCL*, *HMGCS1*, *HSD17B14*, *HSD17B2*, *HSD17B6*, *IAH1*, *INPP1*, *INSIG2*, *ITPKB*, *ITPKC*, *LBR*, *LEP*, *LPIN1*, *LRP2*, *LRP5*, *LYPLA1*, *LYPLA2*, *MBLAC2*, *MBOAT2*, *MBTPS1*, *MGLL*, *MLXIPL*, *MLYCD*, *MSMO1*, *NAA40*, ***NDUFAB1***, *NFE2L1*, *NUDT7*, *OC90*, *ORMDL1*, *OSBPL7*, *OXCT1*, *PC*, *PEX2*, *PGAP2*, ***PIGC***, *PLA2G16*, *PLA2G4A*, *PLAAT5*, *PLCB3*, *PLCD3*, *PLCG2*, *PLCH2*, *PLD3*, ***PNPLA2***, *PPARA*, *PPP6R1*, ***PRDX6***, *PRPF19*, *PRXL2B*, *PSAP*, *PTGES3*, *PTGS2*, *RAB7A*, *RDH13*, *RDH16*, *RUBCNL*, *SCCPDH*, ***SCNN1B***, *SDR16C5*, *SDR42E1*, *SDR42E2*, *SDR9C7*, *SERINC1*, *SGPL1*, *SH3GLB1*, *SIRT2*, ***SMG1***, *SMPDL3A*, *SOAT1*, *SOCS6*, *SOCS7*, *SPART*, *SPHK2*, *SPTLC2*, *SPTSSA*, *SQLE*, *SULT2B1*, *TM7SF2*, *TMEM68*, *TMEM86A*, *TMEM86B*, ***UMOD***	1.59 × 10^−4^
GO:0005975 Carbohydrate metabolic process (N. 52)	*AKT2*, *ATG2A*, *B3GAT3*, *B3GLCT*, *C1QTNF12*, *CHIA*, ***CHID1***, *CHST3*, *CHST6*, *CS*, *DHDH*, ***EDEM3***, *FOXK1*, *FUCA1*, *FUT1*, *FUT2*, *G6PC2*, *GALE*, *GANAB*, *GGTA1*, *GYS1*, *HAS2*, ***HEXB***, *HSD17B14*, *HTR2A*, ***IGF2***, ***INS***, *KCNQ1*, *KL*, *LEP*, *LRP5*, *MDH2*, *NPL*, *NR1D1*, *OVGP1*, *PC*, *PDX1*, *PGM3*, *PLA2G4A*, *PPARA*, *PPP1CA*, *PPP1R3G*, *PYGM*, *RB1CC1*, *SEC1*, ***SIAE***, *SLC3A2*, *SORD*, *ST8SIA4*, *TALDO1*, *TKFC*, *WIPI2*	0.0242

**Table 2:** Genes involved in protein, lipid and carbohydrate metabolic processes in common between QTL regions affecting beef and pork flavor.

*Bos taurus*			Genes	*Sus scrofa*		
Symbol	QTL-ID	BTA		SSC	QTL-ID	Symbol
ABODOR	4837	16	*PIGC*	9	3812	OFFFLAV
JUICE	4838	16	*EDEM3*	9	164889	OVIM
			*PRDX6*	9	3818	OFFFLAV
JUICE	151940	20	*HEXB*	2	5758	JUICE
BEEFOD	4847	25	*ACSM2B*	3	3815	OFFFLAV
			*ACSM3*			
			*ACSM4*			
			*ACSM5*			
			*GDE1*			
			*SCNN1B*			
			*NDUFAB1*			
			*SMG1*			
			*UMOD*			
ABFLAV	4850	29	*SIAE*	9	3818	OFFFLAV
JUICE	4851	29	*DHCR7*	2	164959	JUICE
			*INS*			
			*IGF2*			
			*CHID1*			
			*PNPLA2*			

## Data Availability

Data used for the present paper are available in the Supplementary Tables.

## Supplementary Materials

The following supporting information can be downloaded at: https://www.mdpi.com/article/10.3390/ani16071003/s1, Table S1. List of beef flavor QTLs from Cattle QTL Database. Marker positions were verified by checking Ensembl genome browser (release 115) and *Bos taurus* assembly ARS-UCD2.0 (GCF_002263795.3); Table S2. List of breeds and crosses used for the identification of QTLs associated with beef flavor; Table S3. List of genes located in the autosomal beef flavor QTL regions.

## Abbreviations

The following abbreviations are used in this manuscript:

ACS	Acyl-coenzyme A synthetase
BF	Backfat
BMI	Body mass index
BTA	*Bos taurus*
CoA	Coenzyme A
FA	Fatty acid
GO	Gene Ontology
IMF	Intramuscular fat
Mbp	Million base pair
NEFA	Non-esterified fatty acid
QTL	Quantitative Trait Loci
SNP	Single Nucleotide Polymorphism
SSC	*Sus scrofa*

## References

[1] Sensory Analysis—Vocabulary Reviewed and Confirmed in 2024. https://www.iso.org/standard/38051.html.

[2] Reicks A.L., Brooks J.C., Garmyn A.J., Thompson L.D., Lyford C.L., Miller M.F. (2011). Demographics and beef preferences affect consumer motivation for purchasing fresh beef steaks and roasts. Meat Sci..

[3] Kerth C.R., Miller R.K. (2015). Beef flavor: A review from chemistry to consumer. J. Sci. Food Agric..

[4] Garmyn A. (2020). Consumer Preferences and Acceptance of Meat Products. Foods.

[5] Pogorzelski G., Wozniak K., Polkinghorne R., Poltorak A., Wierzbicka A. (2020). Polish consumer categorisation of grilled beef at 6 mm and 25 mm thickness into quality grades, based on Meat Standards Australia methodology. Meat Sci..

[6] Sakowski T., Grodkowski G., Golebiewski M., Slosarz J., Kostusiak P., Solarczyk P., Puppel K. (2022). Genetic and environmental determinants of beef quality—A Review. Front. Vet. Sci..

[7] Ardicli S., Ardicli O., Ustuner H. (2024). Unraveling the Complexities of Beef Marination: Effect of Marinating Time, Marination Treatments, and Breed. Foods.

[8] Wojtasik-Kalinowska I., Farmer L.J., Hagan T.D.J., Gordon A.W., Polkinghorne R., Pogorzelski G., Wierzbicka A., Poltorak A. (2024). The influence of cooking methods and muscle on beef aroma profile and consumer satisfaction: Insights from volatile compound analysis. Appl. Sci..

[9] O’Quinn T.G., Legako J.F., Woerner D.R., Kerth C.R., Nair M.N., Brooks J.C., Lancaster J.M., Miller R.K. (2024). A current review of U.S. beef flavor II: Managing beef flavor. Meat Sci..

[10] Dinh T.T., To K.V., Schilling M.W. (2021). Fatty acid composition of meat animals as flavor precursors. Meat Muscle Biol..

[11] Di Gregorio P., Grassi G., Perna A.M., Sabia E., Langella E., Di Trana A., Braghieri A. (2025). Genes involved in lipid, carbohydrate and protein metabolic processes located in QTL regions affecting pork meat flavor. Gene.

[12] Hu Z.L., Park C.A., Reecy J.M. (2022). Bringing the Animal QTLdb and CorrDB into the future: Meeting new challenges and providing updated services. Nucleic Acids Res..

[13] Dyer S.C., Austine-Orimoloye O., Azov A.G., Barba M., Barnes I., Barrera-Enriquez V.P., Becker A., Bennett R., Beracochea M., Berry A. (2025). Ensembl 2025. Nucleic Acids Res..

[14] Huang D.W., Sherman B.T., Lempicki R.A. (2009). Systematic and integrative analysis of large gene lists using DAVID bioinformatics resources. Nat. Protoc..

[15] Sherman B.T., Hao M., Qiu J., Jiao X., Baseler M.W., Lane H.C., Imamichi T., Chang W. (2022). DAVID: A web server for functional enrichment analysis and functional annotation of gene lists (2021 update). Nucleic Acids Res..

[16] Alexander L.J., Macneil M.D., Geary T.W., Snelling W.M., Rule D.C., Scanga J.A. (2007). Quantitative trait loci with additive effects on palatability and fatty acid composition of meat in a Wagyu-Limousin F2 population. Anim. Genet..

[17] Gutierrez-Gil B., Wiener P., Nute G.R., Burton D., Gill J.L., Wood J.D., Williams J.L. (2008). Detection of quantitative trait loci for meat quality traits in cattle. Anim. Genet..

[18] Gill J.L., Bishop S.C., McCorquodale C., Williams J.L., Wiener P. (2009). Association of selected SNP with carcass and taste panel assessed meat quality traits in a commercial population of Aberdeen Angus-sired beef cattle. Genet. Sel. Evol..

[19] Allais S., Leveziel H., Payet-Duprat N., Hocquette J.F., Lepetit J., Rousset S., Denoyelle C., Bernard-Capel C., Journaux L., Bonnot A. (2010). The two mutations, Q204X and nt821, of the myostatin gene affect carcass and meat quality in young heterozygous bulls of French beef breeds. J. Anim. Sci..

[20] Gill J.L., Bishop S.C., McCorquodale C., Williams J.L., Wiener P. (2010). Associations between single nucleotide polymorphisms in multiple candidate genes and carcass and meat quality traits in a commercial Angus-cross population. Meat Sci..

[21] Reardon W., Mullen A.M., Sweeney T., Hamill R.M. (2010). Association of polymorphisms in candidate genes with colour, water-holding capacity, and composition traits in bovine M. longissimus and M. semimembranosus. Meat Sci..

[22] Gill J.L., Bishop S.C., McCorquodale C., Williams J.L., Wiener P. (2012). Identification of polymorphisms in the malic enzyme 1, NADP(+)-dependent, cytosolic and nuclear receptor subfamily 0, group B, member 2 genes and their associations with meat and carcass quality traits in commercial Angus cattle. Anim. Genet..

[23] Dang C.G., Cho S.H., Sharma A., Kim H.C., Jeon G.J., Yeon S.H., Hong S.K., Park B.Y., Kang H.S., Lee S.H. (2014). Genome-wide Association Study for Warner-Bratzler Shear Force and Sensory Traits in Hanwoo (Korean Cattle). Asian-Australas J. Anim. Sci..

[24] Lee S.H., Kim S.C., Chai H.H., Cho S.H., Kim H.C., Lim D., Choi B.H., Dang C.G., Sharma A., Gondro C. (2014). Mutations in calpastatin and μ-calpain are associated with meat tenderness, flavor and juiciness in Hanwoo (Korean cattle): Molecular modeling of the effects of substitutions in the calpastatin/μ-calpain complex. Meat Sci..

[25] Aviles C., Pena F., Polvillo O., Barahona M., Campo M.M., Sanudo C., Juarez M., Horcada A., Alcalde M.J., Molina A. (2015). Association between functional candidate genes and organoleptic meat traits in intensively-fed beef. Meat Sci..

[26] Horodyska J., Sweeney T., Ryan M., Hamill R.M. (2015). Novel SNPs in the Ankyrin 1 gene and their association with beef quality traits. Meat Sci..

[27] Mateescu R.G., Garrick D.J., Reecy J.M. (2017). Network Analysis Reveals Putative Genes Affecting Meat Quality in Angus Cattle. Front. Genet..

[28] Leal-Gutierrez J.D., Elzo M.A., Johnson D.D., Hamblen H., Mateescu R.G. (2019). Genome wide association and gene enrichment analysis reveal membrane anchoring and structural proteins associated with meat quality in beef. BMC Genom..

[29] Rezende F.M., Rodriguez E., Leal-Gutierrez J.D., Elzo M.A., Johnson D.D., Carr C., Mateescu R.G. (2021). Genomic Approaches Reveal Pleiotropic Effects in Crossbred Beef Cattle. Front. Genet..

[30] Wyrwisz J., Poltorak A., Zalewska M., Zaremba R., Wierzbicka A. (2012). Analysis of relationship between basic composition, pH, and physical properties of selected bovine muscles. J. Vet. Res..

[31] Kajiya K., Arino M., Koshio A., Minami Y. (2023). Composition and taste of beef, pork, and duck meat and bioregulatory functions of imidazole dipeptides in meat. Sci. Rep..

[32] Vicente F., Pereira P.C. (2024). Pork meat composition and health: A Review of the evidence. Foods.

[33] Watkins P.A., Maiguel D., Jia Z., Pevsner J. (2007). Evidence for 26 distinct acyl-coenzyme A synthetase genes in the human genome. J. Lipid Res..

[34] Singh A., Malla W.A., Kumar A., Jain A., Thakur M.S., Khare V., Tiwari S.P. (2023). Review: Genetic background of milk fatty acid synthesis in bovines. Trop. Anim. Health Prod..

[35] Widmann P., Nuernberg K., Kuehn C., Weikard R. (2011). Association of an ACSL1 gene variant with polyunsaturated fatty acids in bovine skeletal muscle. BMC Genet..

[36] Yu X., Fang X., Xiao H., Zhao Z., Maak S., Wang M., Yang R. (2019). The effect of acyl-CoA synthetase long-chain family member 5 on triglyceride synthesis in bovine preadipocytes. Arch. Anim. Breed..

[37] Murakami Y., Siripanyaphinyo U., Hong Y., Tashima Y., Maeda Y., Kinoshita T. (2005). The initial enzyme for glycosylphosphatidylinositol biosynthesis requires PIG-Y, a seventh component. Mol. Biol. Cell..

[38] Schleinitz D., Kloting N., Lindgren C.M., Breitfeld J., Dietrich A., Schon M.R., Lohmann T., Dreßler M., Stumvoll M., McCarthy M.I. (2014). Fat depot-specific mRNA expression of novel loci associated with waist-hip ratio. Int. J. Obes..

[39] Polla D.L., Edmondson A.C., Duvet S., March M.E., Sousa A.B., Lehman A., Niyazov D., van Dijk F., Demirdas S., CAUSES Study (2021). Bi-allelic variants in the ER quality-control mannosidase gene EDEM3 cause a congenital disorder of glycosylation. Am. J. Hum. Genet..

[40] Reis H.B.D., Carvalho M.E., Espigolan R., Poleti M.D., Ambrizi D.R., Berton M.P., Ferraz J.B.S., de Mattos Oliveira E.C., Eler J.P. (2024). Genome-Wide Association (GWAS) Applied to Carcass and Meat Traits of Nellore Cattle. Metabolites.

[41] Chen J.W., Dodia C., Feinstein S.I., Jain M.K., Fisher A.B. (2000). 1-Cys peroxiredoxin, a bifunctional enzyme with glutathione peroxidase and phospholipase A2 activities. J. Biol. Chem..

[42] Rahaman H., Herojit K., Singh L.R., Haobam R., Fisher A.B. (2024). Structural and functional diversity of the peroxiredoxin 6 enzyme family. Antioxid. Redox Signal..

[43] Liu Y., Wu W.J., Zuo B., Ren Z.Q., Xiong Y.Z. (2011). Polymorphism in coding region of pig PRDX6 gene and its genetic effects analysis. Yi Chuan Hered..

[44] Hepbildikler S.T., Sandhoff R., Kolzer M., Proia R.L., Sandhoff K. (2002). Physiological substrates for human lysosomal beta -hexosaminidase S. J. Biol. Chem..

[45] Gravel R.A., Kaback M.M., Proia R.L., Sandhoff K., Suzuki K., Suzuki K., Valle D.L., Antonarakis S., Ballabio A., Beaudet A.L., Mitchell G.A. (2019). The GM2 Gangliosidoses. The Online Metabolic and Molecular Bases of Inherited Disease.

[46] Lewis C.J., Chipman S.I., Johnston J.M., Acosta M.T., Toro C., Tifft C.J. (2025). Late-onset GM2 gangliosidosis: Magnetic resonance imaging, diffusion tensor imaging, and correlational fiber tractography differentiate Tay–Sachs and Sandhoff diseases. J. Neurol..

[47] Eggen A., Fries R. (1992). An Eco RI restriction fragment length polymorphism at the bovine HEXB locus. Anim. Genet..

[48] Stroud D.A., Surgenor E.E., Formosa L.E., Reljic B., Frazier A.E., Dibley M.G., Osellame L.D., Stait T., Beilharz T.H., Thorburn D.R. (2016). Accessory subunits are integral for assembly and function of human mitochondrial complex I. Nature.

[49] Zhang R., Hou T., Cheng H., Wang X. (2019). NDUFAB1 protects against obesity and insulin resistance by enhancing mitochondrial metabolism. FASEB J..

[50] Zhou J., Tang T., Sun W., Jia X., Wang J., Yu H., Lai S. (2025). NDUFAB1 as a novel regulator of NEFA-induced metabolic dysfunction in bovine adipocytes. Animals.

[51] Zhu J., Wang Y., Su Y., Zheng M., Cui H., Chen Z. (2024). RNA sequencing identifies key genes involved in intramuscular fat deposition in chickens at different developmental stages. BMC Genom..

[52] Devuyst O., Olinger E., Rampoldi L. (2017). Uromodulin: From physiology to rare and complex kidney disorders. Nat. Rev. Nephrol..

[53] Trudu M., Schaeffer C., Riba M., Ikehata M., Brambilla P., Messa P., Martinelli-Boneschi F., Rastaldi M.P., Rampoldi L. (2017). Early involvement of cellular stress and inflammatory signals in the pathogenesis of tubulointerstitial kidney disease due to UMOD mutations. Sci. Rep..

[54] Florens N., Calzada C., Lyasko E., Juillard L., Soulage C.O. (2016). Modified lipids and lipoproteins in chronic kidney disease: A new class of uremic toxins. Toxins.

[55] Zeravica R., Ilincic B., Buric D., Jakovljevic A., Crnobrnja V., Ilic D., Papuga M.V. (2024). Relationship between serum uromodulin as a marker of kidney damage and metabolic status in patients with Chronic Kidney Disease of non-diabetic etiology. Int. J. Mol. Sci..

[56] Prabhu A.V., Luu W., Li D., Sharpe L.J., Brown A.J. (2016). DHCR7: A vital enzyme switch between cholesterol and vitamin D production. Prog. Lipid Res..

[57] Li Z., Hu T., Li R., Li J., Wang Y., Li Y., Lin Y., Wang Y., Jiani X. (2023). Effect of DHCR7 on adipocyte differentiation in goats. Anim. Biotechnol..

[58] Zhou C., Gui W., Zhu W., Zhao H., Wu D., Wu F., Wang G., Lin X. (2025). Paradoxical regulation of IGF2 in promoting lipid metabolism in adipose tissues. Commun. Biol..

[59] Gaunt T.R., Cooper J.A., Miller G.J., Day I.N., O’Dell S.D. (2001). Positive associations between single nucleotide polymorphisms in the IGF2 gene region and body mass index in adult males. Hum. Mol. Genet..

[60] Criado-Mesas L., Ballester M., Crespo-Piazuelo D., Castello A., Benitez R., Fernandez A.I., Folch J.M. (2019). Analysis of porcine IGF2 gene expression in adipose tissue and its effect on fatty acid composition. PLoS ONE.

[61] Berkowicz E.W., Magee D.A., Sikora K.M., Berry D.P., Howard D.J., Mullen M.P., Evans R.D., Spillane C., MacHugh D.E. (2011). Single nucleotide polymorphisms at the imprinted bovine insulin-like growth factor 2 (IGF2) locus are associated with dairy performance in Irish Holstein-Friesian cattle. J. Dairy Res..

[62] Huang Y.Z., Wang J., Zhan Z.Y., Cao X.K., Sun Y.J., Lan X.Y., Lei C.Z., Zhang C.L., Chen H. (2013). Assessment of association between variants and haplotypes of the IGF2 gene in beef cattle. Gene.

[63] Norton L., Shannon C., Gastaldelli A., DeFronzo R.A. (2022). Insulin: The master regulator of glucose metabolism. Metabolism.

[64] Cerk I.K., Wechselberger L., Oberer M. (2018). Adipose triglyceride lipase regulation: An Overview. Curr. Protein Pept. Sci..

[65] Cui H., Meng Q., Qu Y., Liu H., Feng C., Wang H., Liu Y., Zan L., Li N. (2011). Two novel missense mutations in bovine ATGL gene and their association with economic traits in Qinchuan cattle. Afr. J. Biotechnol..

[66] Qiu Y., Ding R., Zhuang Z., Wu J., Yang M., Zhou S., Ye Y., Geng Q., Xu Z., Huang S. (2021). Genome-wide detection of CNV regions and their potential association with growth and fatness traits in Duroc pigs. BMC Genom..

[67] Lee S., Jo K., Park M.K., Choi Y.S., Jung S. (2025). Role of lipids in beef flavor development: A review of research from the past 20 years. Food Chem..

[68] Zhang T., Wang T., Gao Y., Sheng J., Rushdi H.E., Li W., Sun Y., Fu T., Lin F., Gao T. (2025). Flavor, lipid, and transcriptomic profiles of chinese wagyu beef cuts: Insights into meat quality differences. Foods.

[69] Fu Y., Cao S., Yang L., Li Z. (2022). Flavor formation based on lipid in meat and meat products: A review. J. Food Biochem..

